# Medical findings and symptoms in infants exposed to witnessed or admitted abusive shaking: A nationwide registry study

**DOI:** 10.1371/journal.pone.0240182

**Published:** 2020-10-13

**Authors:** Ingemar Thiblin, Jacob Andersson, Knut Wester, Johan Wikström, Göran Högberg, Ulf Högberg

**Affiliations:** 1 Department of Surgical Sciences, Uppsala University, Uppsala, Sweden; 2 Department of Clinical Medicine K1, University of Bergen, Bergen, Norway; 3 Department of Surgical Sciences, Uppsala University, Uppsala, Sweden; 4 Private Practice, Stockholm, Sweden; 5 Department of Women’s and Children’s Health, Uppsala University, Uppsala, Sweden; 6 Department of Epidemiology and Global Health, Umeå University, Umeå, Sweden; Technion - Israel Institute of Technology, ISRAEL

## Abstract

**Background:**

Many physicians regard the combination of encephalopathy, subdural haemorrhage (SDH), retinal haemorrhage (RH), rib fractures, and classical metaphyseal lesions (CML) as highly specific for abusive head trauma (AHT). However, without observed abuse or other criteria that are independent of these findings, bias risk is high.

**Methods:**

Infants subjected for examination under the suspicion of maltreatment during the period 1997–2014 were identified in the National Patient Registry, International Classification of Diseases (ICD-10 SE). The medical records were scrutinized for identification of cases of witnessed or admitted physical abuse by shaking. The main outcome measures were occurrence of SDH, RH, fractures and skin lesions.

**Results:**

All identified 36 infants had been shaken, and for 6, there was information indicating blunt force impact immediately after shaking. In 30 cases, there were no findings of SDH or RH, rib fractures, or CMLs. Six infants had finding(s) suggestive of physical abuse, two with possible acute intracranial pathology. One infant with combined shaking and impact trauma had hyperdense SDH, hyperdense subarachnoid haemorrhage, suspected cortical vein thrombosis, RH, and bruises. Another infant abused by shaking had solely an acute subarachnoid haemorrhage. Both had pre-existing vulnerability. The first was born preterm and had non-specific frontal subcortical changes. The other had bilateral chronic SDH/hygroma.

**Conclusions:**

The present findings do not support the hypothesis that acute SDH or RH can be caused by isolated shaking of a healthy infant. However, they do suggest that abuse by shaking may cause acute intracranial haemorrhage with RH in infants with certain risk factors.

## Introduction

In 1971, paediatric neurosurgeon Norman Guthkelch proposed that subdural haematoma in infants and toddlers without evidence of blunt force trauma may be caused by a whiplash mechanism arising from forces elicited through shaking [1]. The hypothesis was partly based on a whiplash study in rhesus monkeys subjected to a single acceleration inertial load of 600 G [2]. Three years later, radiologist John Caffey described the combination of subdural haematoma (SDH), retinal haemorrhages (RHs), and clinical signs of brain injury as highly suggestive for whiplash shaking [3]. Kleinman et al. suggested that the thoracic compression exerted during violent shaking was associated with rib fractures, especially posteriorly [4], and that the torsional and tractional forces resulted in the classic metaphyseal lesions (CMLs) known as “bucket-handle” and “corner” fractures [5]. This collective of putative signs of abuse exposure was designated as shaken baby syndrome (SBS), which was changed in 2009 to abusive head trauma (AHT) to include blunt force trauma [6]. For detection of SBS/AHT, a common tool is the Duhaime algorithm, which classifies as “suspicious, but not presumptive for inflicted injury” the failure of a caretaker to provide a plausible trauma history that could account for such findings [7]. According to another algorithm, this collective of signs makes a diagnosis of abuse “highly probable” [8].

Biomechanical research has addressed the forces derived from shaking. Ommaya et al. [9] questioned the reasoning of Caffey [3] and Guthkelch [1], who hypothesised shaking as a mechanism for SDH, RH, and encephalopathy in his study of rhesus monkeys subjected to a single severe inertial load [2]. Ommaya et al. found that the forces causing subdural haemorrhage in the experimental model in that study could only be produced by an impact load that not even the most violent shaking could give rise to. Two reports on use of biofidelic dolls offered similar conclusions [10, 11]. One biofidelic study challenged these findings, however, by generating higher forces if certain parameters were changed [12]. Those authors concluded that it is not possible to categorically rule out shaking as a cause in lethal brain injury.

Duhaime’s algorithm also has been employed in clinical observational studies, case series, and cross-sectional studies of establishing an abuse diagnosis. If SDH is detected in an infant in the absence of a witnessed accident or disease known to cause SDH, abuse can be inferred from the history given by the parents or caregivers. In 2011, Maguire et al. proposed as the highest ranking for a diagnosis of AHT “abuse confirmed at case conference or civil, family, or criminal court proceeding, or admitted by the perpetrator or independently witnessed” [13]. Under this strategy, a caregiver statement of absence of trauma was used as a diagnostic factor in two of three SDH cases [14], a clear bias toward circular reasoning in assessing the association of shaking and SDH. Several studies have addressed this problem with circular bias in AHT research [14–16]. It was the main methodological flaw identified in the Swedish Agency for Health Technology Assessment and Assessment of Social Services authority’s systematic review of the role of SDH, RH, or encephalopathy in medical investigations of suspected traumatic shaking [17].

This review has been subjected to much criticism with responses from the expert group and others [18–23]. To our understanding the conclusions from the SBU systematic review are solid, while the need of more research was highlighted.

In their review, the authority used the PIRO format (population, intervention, reference standard, outcome) [17]. Admitted or witnessed traumatic shaking was the reference standard test to evaluate the diagnostic accuracy that shaking causes SDH, RH, or encephalopathy. Two studies [24, 25] demonstrated that shaking was associated with SDH and RH, but the circumstances under which confessions were obtained were not described in detail, and the studies were assessed as being of moderate quality [17]. Confessions in police interrogation or court might be flawed evidence in support of a cause–effect association between shaking and SDH, RH, or encephalopathy [26].

In this study, we further tested the hypothesis that shaking causes SDH, RH, encephalopathy, rib fractures, or CMLs, using a definition of physical abuse by shaking that is independent of medical findings or symptoms, i.e., not compromised by circularity. To address this question, we evaluated symptoms and medical findings in infants subjected to admitted or witnessed shaking, with or without blunt force head impact.

## Materials and methods

### Study design

The design is a case series. Infants (0–365 days) who underwent examination with suspicion of maltreatment during 1997 to 2014 were identified in the Swedish National Patient Register by ICD-10 codes (Swedish version of International Classification of Diseases 10^th^ version): Z03.8K (observation for suspected maltreatment), Y07.9 (maltreatment and neglect by unspecified perpetrator), T74.1 (physical abuse, confirmed), and Y06 (neglect and abandonment). Data were linked to the Swedish Medical Birth Registry [27]. The medical records were requested from the paediatric departments in Sweden.

### Inclusion criteria

The reference test was chosen to avoid bias by circular reasoning, as suggested by Högberg et al. [14]. Witnessed shaking was defined as shaking having been observed directly or filmed (e.g., by web cam or smartphone). Admitted shaking was defined as a spontaneously delivered history of shaking before or in direct connection with seeking care before diagnostic procedures such as computed tomography (CT), magnetic resonance imaging (MRI), or fundoscopy were performed. In the case of admitting shaking before seeking care, e.g., to social services, the history of shaking had to be maintained when seeking care at the hospital. Cases with a history of combined shaking and blunt force impact trauma were included to meet the broadened criteria of exposure for abusive head trauma published by the American Academy of Pediatrics in 2009 [6]. No exclusion criteria were applied with respect to details of the shaking event(s), such as perceived force of shaking, number of shaking cycles, number of shaking events, or relationship between the offender and the witness.

### Exclusion criteria

Cases with shaking after collapse for resuscitation purposes were excluded. Cases with reported blunt head trauma of any kind but lacking a history of shaking were not included.

### Participants

A total of 337 infants with any maltreatment diagnosis were identified, and medical records could be retrieved for 257 (76%). Missing cases by region were as follows: of 80 cases from the southern region, 16 (20%) were missing; of 41 cases from the southeast region, 12 (29%) were missing; 3 (7%) of 41 cases from the western region were missing; 25 of 68 cases (37%) were missing from the Uppsala-Örebro region; 23 of 88 cases (26%) from the Stockholm region were missing; and 1 of 17 cases (6%) from the northern region could not be found. In one case, the region could not be identified.

**Fig 1:**
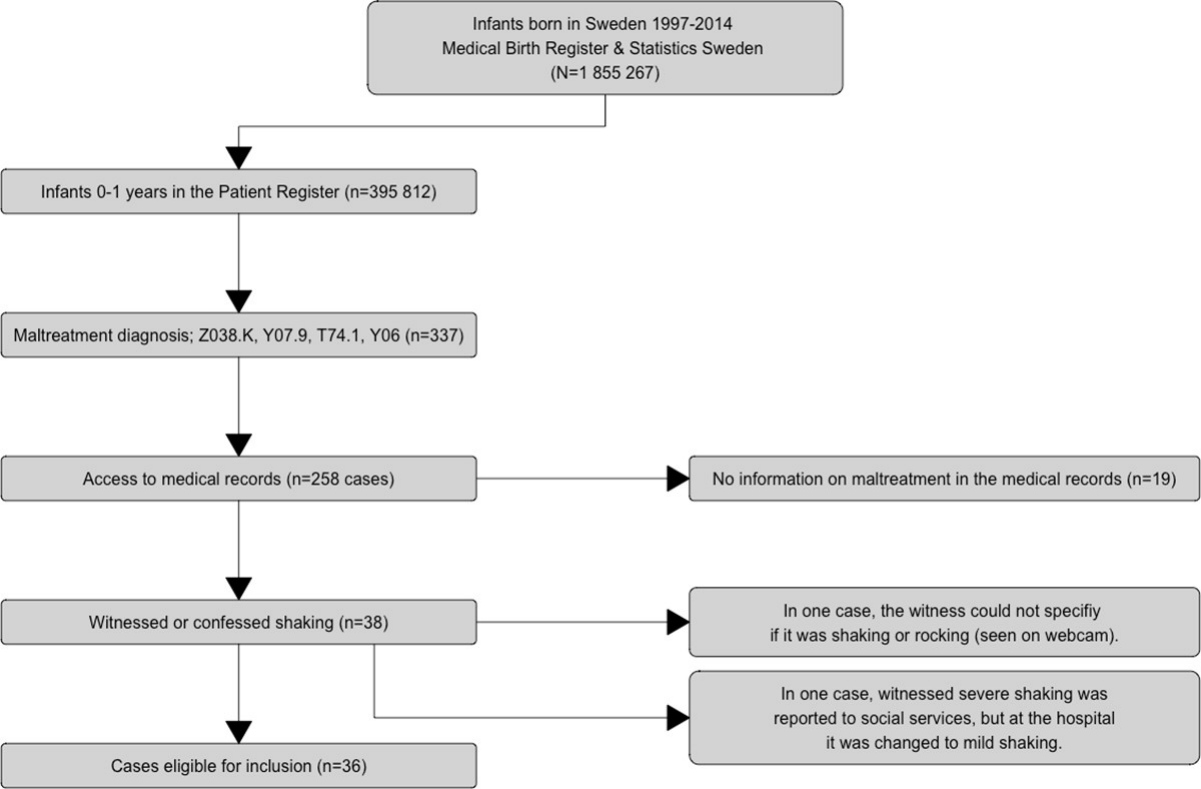
Selection of included cases.

In 19 cases, the reason for seeking care was related to different diseases, with no information suggestive of maltreatment. These cases were regarded as wrongly coded. In the remaining 239 cases, there was a suspicion of maltreatment for various reasons. After application of inclusion and exclusion criteria, 36 cases remained. Fig 1 provides a flow chart of the case selection.

#### Covariates

Data regarding age (in weeks), sex, gestational week, birth weight, and neonatal or infant morbidity at the time of the consultation were retrieved from the Swedish Medical Birth Registry. Raw data from CT were provided in two cases. An experienced neuroradiologist (JW) evaluated these examinations.

### Outcome

Outcomes were frequency of findings from neuroimaging, fundoscopy, whole-body x-ray, and clinical examination, and symptoms registered at clinical examination.

### Statistics

An *ad hoc* test for statistical significance of the observed disproportion in sex was performed by applying the Z-statistics, assuming a null hypothesis value of 50% and employing MedCalc software [28].

## Ethics approval and consent to participate

The Regional Ethical Review Board in Uppsala approved the study (2014-11-19 No 383). This committee approved a waiver of informed consent, considering that the research database contained only coded data. Register linkage was provided by the National Board of Health and Welfare. Approval for ordering medical records of those with a maltreatment diagnosis was obtained by the Regional Ethical Review Board in Uppsala (2015-11-18 No 383/2) and by having the personal ID of the case medical records retrieved after each hospital’s approval according to Swedish Public Law and Privacy Act (2009:400). The medical records were coded during the analysis. The code key was kept separate in a locked computer. The researchers had no access to the personal ID during the analysis. Dissemination of the results to the study participants was not possible.

## Results

### Circumstances defining AHT

Criteria for abuse by shaking were met in 36 cases, 33 of them witnessed. In 21 cases, the mother was the witness and the father the perpetrator; in five cases, the father was the witness and the mother was the perpetrator. One case was filmed by the father with the mother as the perpetrator, five cases by strangers or neighbours, and one case by a person whose relation to the perpetrator was not recorded. In three cases, the caregiver spontaneously admitted the shaking act when seeking care. In one of the admitted cases (with the father as perpetrator), the partner also witnessed the shaking event.

### Reason for seeking care

The reason for seeking care was concern about possible injury in 35 cases and about seizures in one case.

### Demographics

**Fig 2:**
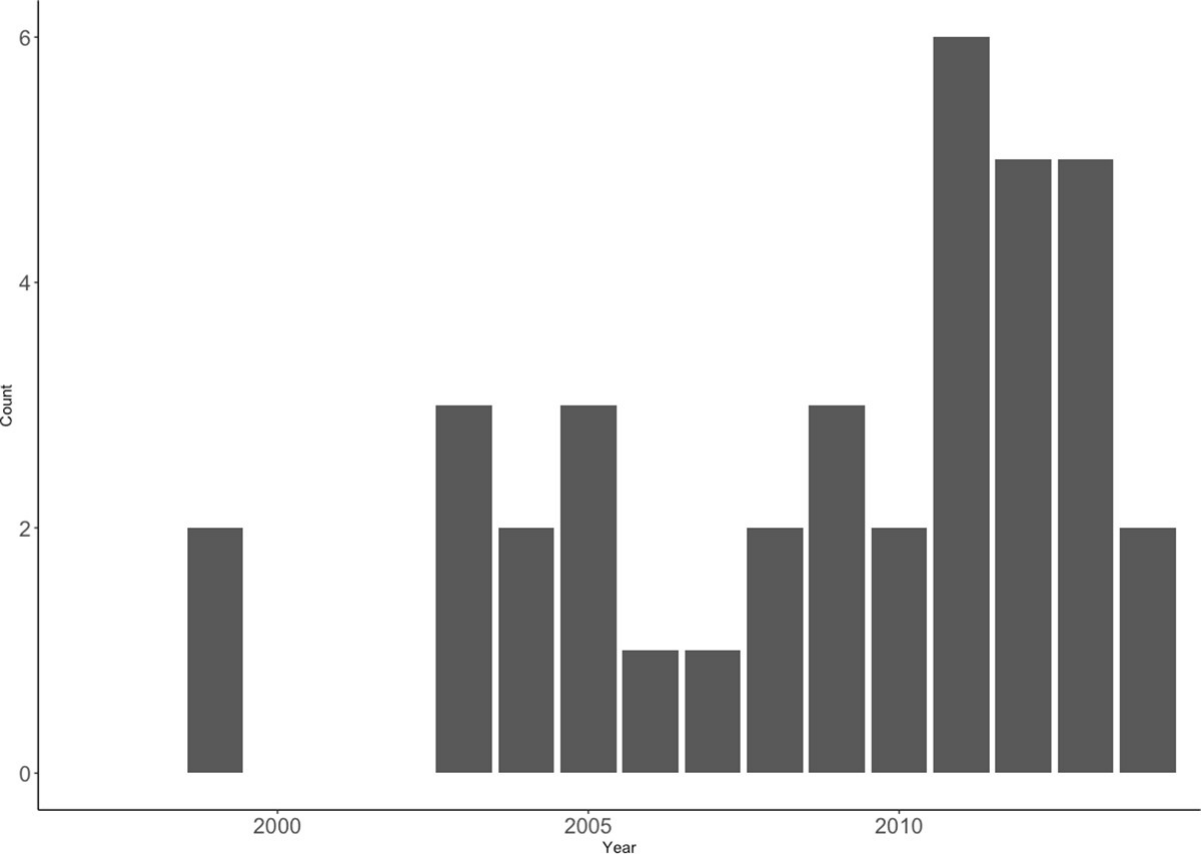
Case count per year during the study period, 1997–2014.

There were 15 boys (42%) and 21 girls (Z-statistic = 0.960; p = 0.34), with a mean age of 135 days and a median age of 104 days (range, 18–337 days). Case rates increased towards the end of the study period (Fig 2).

### Diagnostic procedures

**Fig 3:**
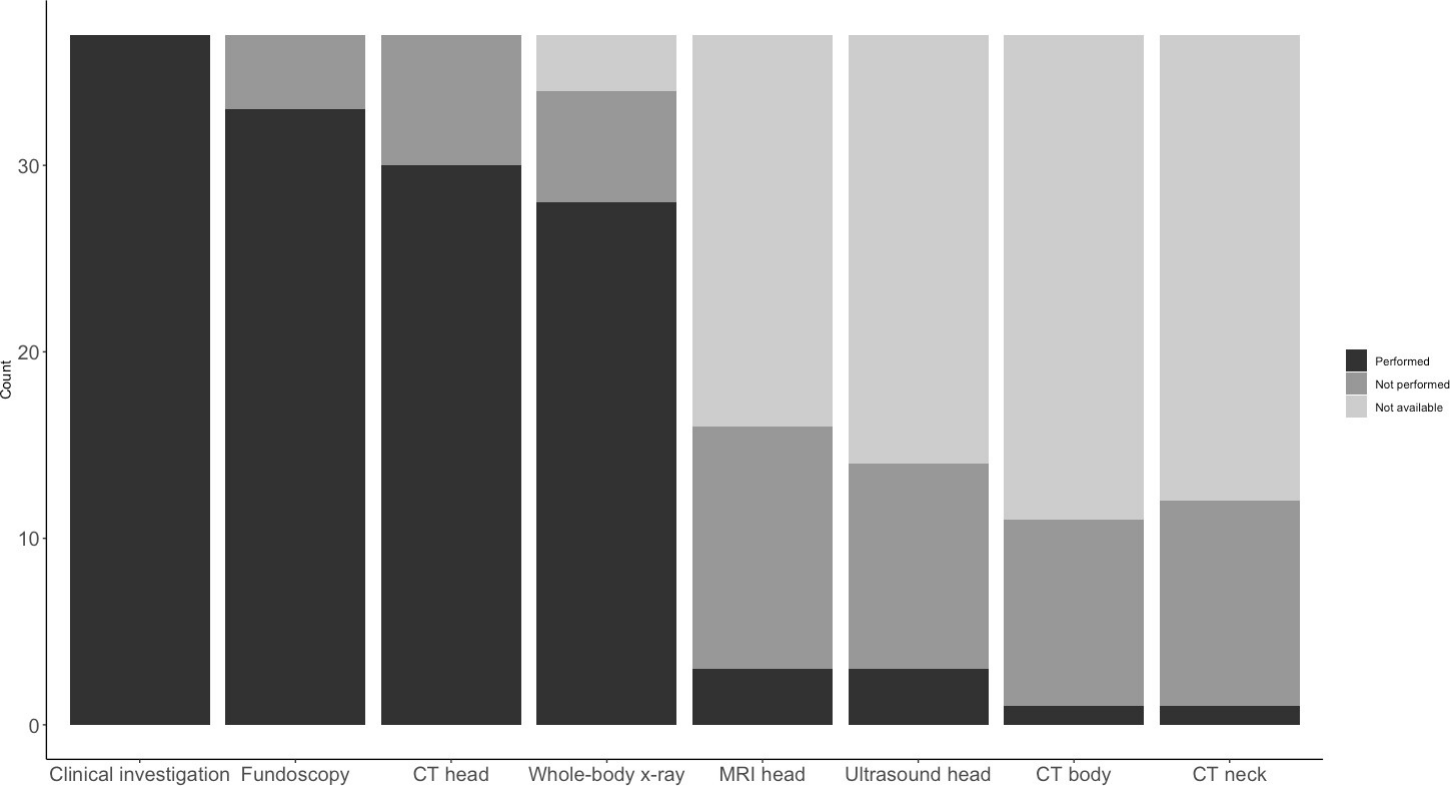
Diagnostic procedures in 36 infants subjected to witnessed shaking with or without blunt force head trauma. “Not available” signifies that it was unclear if the procedure was performed or not.

Clinical examination, including inspection of the entire body, was documented in all 36 cases, and fundoscopy was performed and documented in 32 (89%). Skull/brain CT was performed in 29 (81%), MRI in 3 (8%), and ultrasound of the head in 2 (6%). Five infants underwent no brain imaging. Full-body x-ray examination was performed in 27 (75%) of the cases. In one case, there was only a whole-body x-ray, and in another, only fundoscopy in addition to clinical examination. The diagnostic procedures are summarized in Fig 3.

### Documented physical violence

In 16 cases, the shaking was described as “forceful” and in one as “hysterical”. In the remaining cases, shaking was mentioned without any modifier with respect to force. In 11 cases, shaking on two or more occasions was mentioned, with five of those in the group where forceful shaking was described.

In two cases, there was a clearly stated deliberate back head impact in connection with shaking. In four additional cases, there was a statement of throwing the infant in connection with shaking. The part of the body that was exposed to impact was not reported in any of these cases. In yet another case, the infant was kicked into a wall, but there was no information about a temporal relationship between this event and shaking. Details of the physical violence are provided in S1 Table.

### Findings and symptoms

In 30 cases, no medical findings or symptoms were reported. All infants underwent clinical examination. One of them did not undergo any further diagnostic procedures. Of these 30, the remaining 29 infants were examined by ancillary diagnostics, as described, including 20 who had whole-body x-ray, CT/MRI, and fundoscopy; five who underwent CT/MRI and fundoscopy; one who had head ultrasound and fundoscopy; two who had whole-body x-ray only; and one who had fundoscopy only. Twelve of the infants without any reported symptoms or findings and who had been examined by CT/MRI and fundoscopy had a history of forceful shaking.

**Fig 4:**
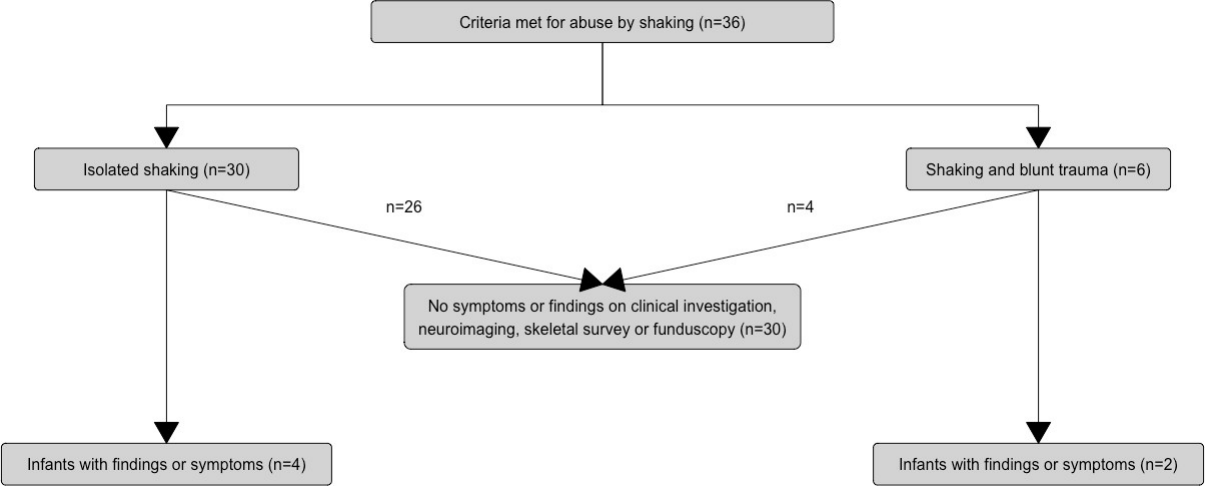
Infants with findings/symptoms with respect to type of violence exposure.

Four infants with a history of isolated shaking and two infants with combined shaking and blunt force trauma had medical findings and/or symptoms. Fig 4 shows the proportions of infants with these findings in the context of the type of violence exposure.

Three infants had skin lesions (cases 7, 8, 28). In one case, these consisted of reddish marks on the shoulders consistent with handgrip, according to the examining paediatrician (case 8).

Any degree of reduced consciousness was reported in two cases (cases 8, 9). Vomiting in connection with shaking was reported in three cases (cases 9, 16, 20). Seizures in connection with shaking were reported in one case (case 9).

Two infants (6% of the 32 with brain imaging examination) had intracranial subdural fluid collections described as acute or chronic haemorrhage. One of these infants had a history of combined shaking and blunt force impact (case 8), and the other had a history of shaking only (case 9). Both were boys, and these cases are addressed separately below.

#### Case 8

**Fig 5:**
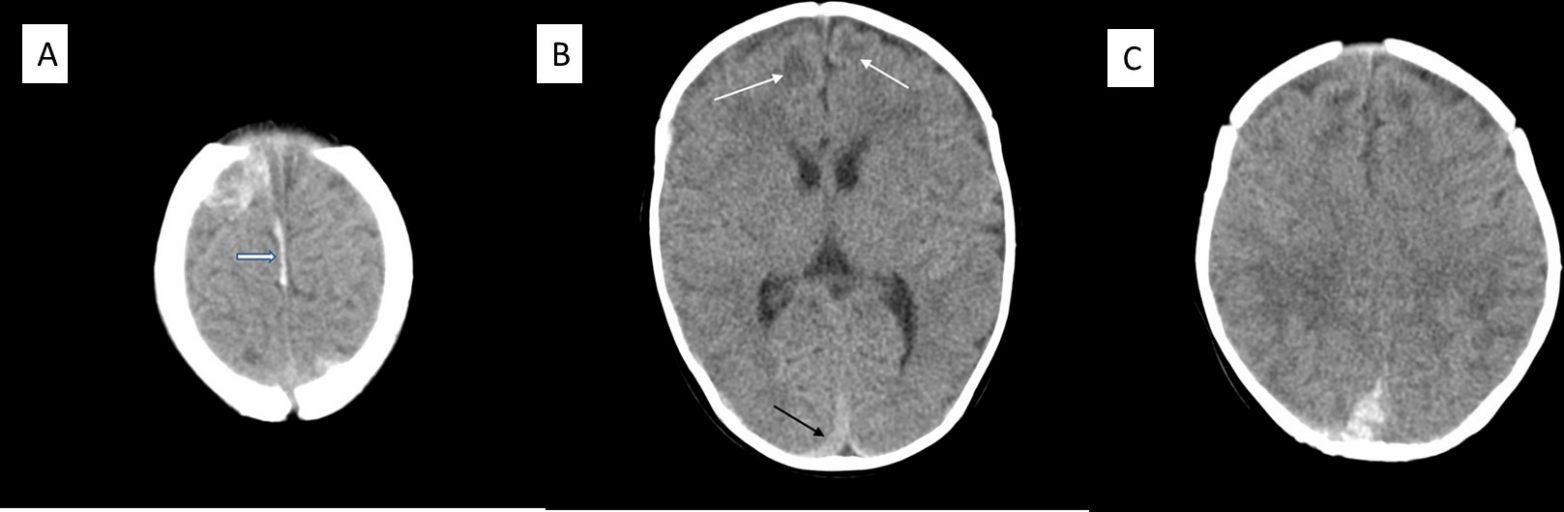
CT images of cases 8. There is acute subarachnoid blood in bilateral sulci over the vertex (A) and suspected cortical venous thromboses (arrow on one of them in A). There is a thin chronic subdural hematoma in the right frontotemporal region, and a localized fresh subdural hematoma in the right parietal region (black arrow in B). There is a small amount of fresh blood to the right of the confluens sinuum (C), probably extraaxially. In addition, there are symmetrical low attenuating parenchymal changes of unspecific appearance (white arrows in B).

According to the original radiological statement, the infant in case 8 (age 11 weeks, preterm week 33) had acute SDH and possible subarachnoid haemorrhage, possible cortical vein thrombosis, and possible small frontal unilateral chronic SDH/hygroma. According to our re-evaluation, there was a small amount of fresh subarachnoid blood bilaterally. There was a thin chronic SDH in the right frontotemporal region, and a localized up to 9 mm thick fresh SDH in the right parietal region. There were two suspected cortical venous thromboses. Additionally, there were symmetrical low-attenuating changes in the deep white matter in the frontal and parietal lobes. These were interpreted as non-specific and of uncertain age. CT findings are illustrated in Fig 5A–5C. This infant underwent a brain ultrasound examination at 4 days of age that demonstrated absence of intracranial haemorrhage or any other focal alteration.

#### Case 9

**Fig 6:**
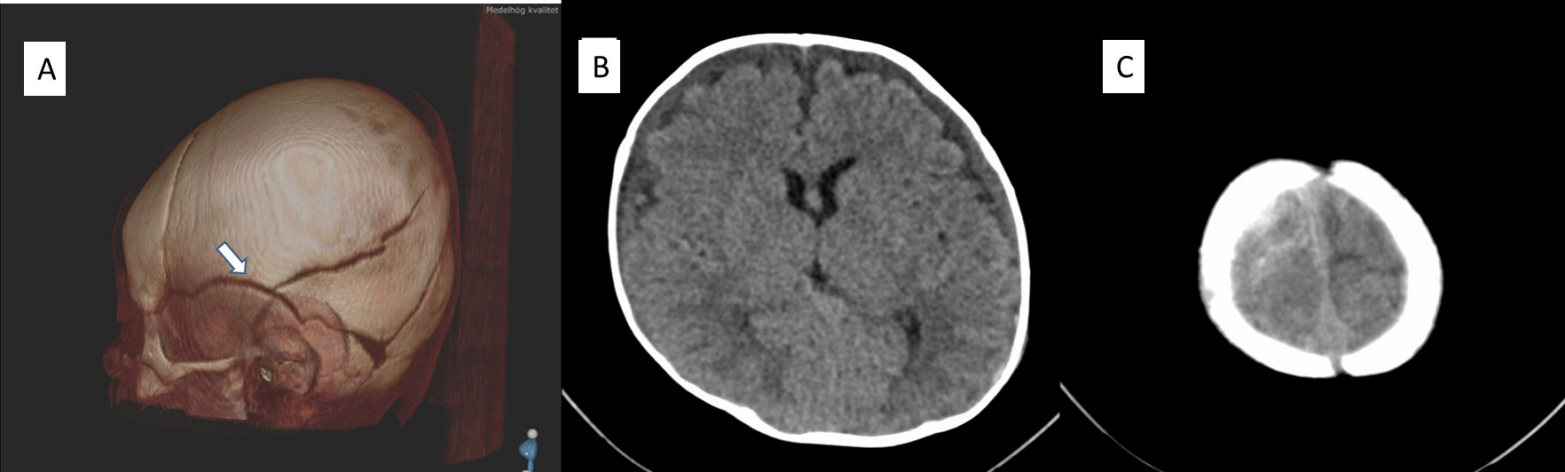
CT images of case 9. There is a temporoparietal fracture of uncertain age on the left side (arrow in A, 3D reconstruction with volume rendering technique). There are bilateral subdural fluid collections compatible with chronic subdural hematomas (B). There is a small amount of fresh subarachnoid blood bilaterally (C).

According to the original radiological statement, the infant in case 9 (age 17 weeks, term) had thin bilateral acute subarachnoid haemorrhage, bilateral chronic SDH, and parietal and temporal fractures in healing stage. According to our re-evaluation, there was a fracture with minimal displacement in the left temporal and parietal bone. The age was not possible to determine, but it could have been recent at the time of examination. There were bilateral extracerebral fluid collections with low attenuation but higher than that of cerebrospinal fluid, and with medial dislocation of superficial vessels, suggesting a chronic SDH rather than enlarged subarachnoid spaces. There was fresh subarachnoid blood bilaterally. There was a small blood clot, probably extra-axial, to the right of the confluens sinuum. CT findings are illustrated in Fig 6A–6C.

*Retinal haemorrhage*. One infant with combined shaking/impact had RHs, described as extensive bilateral (case 8). Another infant, age 3 weeks and without intracranial pathology at radiology, had two RHs in one eye (case 36).

*Skeletal injuries*. One infant (case 20) had a healing clavicular fracture and another infant a possible earlier temporal/parietal facture, described as sclerotic bone (case 28). Rib fractures or classic metaphyseal “bucket-handle and “corner” fractures were not reported in any infant.

### Vulnerability factors

None of the 36 infants was multiple birth. Three were small for gestational age (<25th percentile), and all three were in the group without any symptoms or findings.

Two infants were preterm, born at week 36 and week 33 (cases 3 and 8), respectively. One of those (case 8) had intracranial pathology with a possible temporal relation to the admitted act of abuse. The other preterm infant had no findings or symptoms.

One of the preterm infants (case 8) was treated for neonatal icterus and had episodes of impaired oxygen saturation at age 4 days that was successfully treated with theophylline. The head circumference was measured at three occasions, at ages 9 days, 14 days, and 25 days, and showed a gradual increase from -1.5 standard deviations to slightly above normal.

Other conditions during the neonatal period were laryngospasm on one occasion (case 2), cephalohaematoma (case 14), and damage to the facial nerve (case 12). These three infants were in the group without any medical findings or symptoms.

## Discussion

### Principal findings

In contrast to earlier studies in which shaking or combined shaking/blunt trauma was wholly or partly inferred from SDH, RH, seizures, apnoea, and long bone fractures, we found no strong association between such findings and shaking with or without blunt force impact. None of the infants with reported isolated shaking had any of these findings. However, one of the infants subjected to isolated shaking had an acute subarachnoid haemorrhage. This infant also had a hypodense subdural effusion and skull fractures of unclear age (Fig 6A–6C), suggesting increased vulnerability related to blunt trauma. Another infant with intracranial pathology (discussed further below) in connection with admitted abuse by shaking and possible blunt head trauma at age 11 weeks had a history of prematurity (week 33) and increasing head size until the last notation at age 25 days.

For 30 infants, no findings or symptoms were reported. The shaking was described as forceful/hysterical in 13 of these cases (12 examined with neuroimaging and fundoscopy); one was filmed, and three were observed by nonrelated witnesses. None of the 27 infants who underwent a full-body x-ray had rib fractures or CMLs. Thus, no infant subjected to shaking with or without blunt force trauma and without any possibly predisposing factors had any of the findings regarded as highly specific for AHT.

The brain imaging of the premature infant born at week 33 was restricted to CT. This imaging showed straightforward signs of thin hyperdense bilateral SDHs, small subarachnoid haemorrhages and possible cortical vein thrombosis and symmetrical frontal bilateral hypodense subcortical areas. These descriptive diagnostics are consistent with some alternative aetiologies. The symmetrical low-attenuating changes in the deep white matter in the frontal lobes were non-specific and of uncertain age. The many possible causes include hypoxic–ischemic changes, toxic or metabolic disturbances, or previous infection [29]. Subcortical lesions in infants have been assumed to be of traumatic origin and suggestive of abuse in neuroimaging studies [30–33]. However, neuropathological data do not support direct mechanical forces of trauma as the cause of such findings, and instead point to impaired venous drainage that may or may not follow trauma [34]. This infant had an ultrasound brain examination during the first days of life that revealed no intracranial haemorrhage. However, this method has limited sensitivity for detecting a small SDH in preterm neonates [35], for which reason the possibility of birth-related SDH with re-bleeding as a differential cannot be ruled out. Despite uncertainties about the mechanism(s) behind the intracranial pathology, the temporal relationship between abuse by shaking and possible blunt force and at least partly acute medical findings suggests that AHT related pathology was present against a backdrop of pre-existing pathology of unclear origin. Although the mechanism behind the findings is unclear, tearing of bridging veins seems unlikely, considering the limited volume of the subdural blood collection.

Many studies have yielded extremely high specificity and positive predictive values of SDH and RH for AHT [17]. However, these values were based on circular reasoning and other methodological flaws [17]. The study design of the current study does not allow for conclusions regarding the specificity of medical findings for AHT. However, we identified no intracranial pathology assumed to be specific for abuse. Acute SHD with or without RH in healthy infants can be caused by head impact from low-height falls that do not result in bruises or fractures [36]. To our knowledge, extensive RH has not been described in the absence of intracranial pathology such as SDH, subarachnoid haemorrhage, or hygroma. The only infant in the present study who had extensive RH also had intracranial pathology, including possible cortical vein thrombosis, a condition associated with RH in older children and adults [37, 38]. As far as we know, no systematic studies have addressed RH in infants with cortical venous thrombosis. The infant with two RHs and no acute intracranial pathological changes was only 3 weeks old, so the haemorrhage could possibly have arisen during birth. This infant was vaginally delivered, and up to 33% of babies delivered by unassisted vaginal birth have RHs that usually resolve by age 6 weeks but can persist longer [39].

Skin lesions were recorded in three infants. As always, such lesions must be interpreted in relation to the given context. In the present case series, reddish skin/bruises were described in two cases and soft tissue swelling in a third. In each infant, the skin lesions were consistent with the history of abuse, corroborating the accuracy of the described history of abuse.

In our case series of admitted or witnessed shaking, 27 infants were examined with full-body x-ray and had no findings of rib fracture or CMLs. Thus, our results do not support the hypothesis that rib fractures appear because of thorax compression or CMLs from acceleration and deceleration forces during shaking, as Kleinman et al. had proposed [4].

The proportion of males was 42% in the present study. This finding contrasts with earlier reports in which AHT was inferred from symptoms and intracranial and ocular pathology, and the proportion of males was usually 60% to 80%. For instance, in pooled cases from two studies reporting AHT corroborated by confessions under police interrogation, the proportion of boys was 73% [24, 25]. One possible explanation for this difference in sex distribution could be the presence of cases of benign external hydrocephalus (BEH), a condition with a marked overrepresentation of boys [40–43], that were misdiagnosed as AHT. Spontaneous bleeding is reported to appear in close to 10% of infants with BEH [43]. The potential conflation of BEH for AHT leaves the possibility that false confessions from coercion during police interrogation [26, 44, 45] could explain the overrepresentation of boys in studies reporting confessed abuse.

In view of the Bradford criteria of specificity and temporality, our findings on abusive shaking highlight possible fallacies that are applicable to using the Duhaime algorithm [7] in clinical practice for diagnosing infant abuse (that findings from neuroimaging, x-ray, or fundoscopy enable the clinician to determine what, how, and when an event happened). Possible weaknesses of using technoscience in detecting infant abuse while overlooking clinical history and examination have been addressed [46]. This oversight might have been an origin of the AHT epistemology, deriving from true case observations supporting the Duhaime algorithm, but then becoming biased by circular reasoning [15] that disregarded other causes of such findings [47, 48].

### Strengths and limitations

The strength of the present study is the national coverage over a fairly long period of 18 years, making it possible to identify several cases fulfilling strict criteria of abusive head trauma not compromised by circular bias. Given the rarity of witnessed or spontaneously admitted infant abuse, the cases had to be identified retrospectively. As is always the case, the retrospective study design has its limitations. The quality of the information in the medical records is far from uniform with respect to descriptions of the violent act and to the diagnostic procedures. Most cases were identified by a statement of witnessed abuse, with the partner as the witness. The description of the abuse in such a statement may be exaggerated or perhaps even fabricated if the partners are in an ongoing conflict. The increase over time in abuse cases witnessed by a partner was coincident with an information campaign about the dangers of shaking a baby [49]. Thus, it is possible that observations of relatively “gentle shaking” fuelled concern about possible injuries in a number of cases involving the partner-witness group. In four cases witnessed by an unrelated person or admitted, there were no statements regarding the force of the shaking, making it possible that the shaking was of limited force in these cases, as well.

In cases of abuse witnessed by unrelated observers or documented by filming, shaking that exceeded normal infant rocking was quite likely to have occurred. However, objective measures of the applied forces were not possible for obvious reasons.

The retrospective design also resulted in failure to retrieve medical records for all identified cases. The reason for turning down requisitions is unclear, but the rather pronounced differences in these gaps among different regions in Sweden, ranging from 6% to 37%, indicate that the reluctance to contribute to research in the area of child abuse is stronger in some regions. However, by our review of 258 records out of 337 it is unlikely that detection bias influenced the results. Thus, we believe that our results have a fair generalizability.

We have previously reported that 14% (n = 43) of SDH cases in infancy had also maltreatment diagnosis [47]. Two of these cases fulfilled the case definition of admitted or witnessed shaking or blunt force head trauma in the present study, indicating that AHT was inferred by interpretation of medical findings in at least 41 of the 43 cases. The fact that different strategies for defining AHT identifies different infants as subjected to AHT emphasizes the need for considering the consequences of selection on the generalizability of the results in AHT studies.

### Clinical implications

To our knowledge, this is the first study assessing the association of SDH and RH from prior witnessed or admitted physical abuse by shaking. One previous study of witnessed abusive shaking (n = 21) among 90 cases of witnessed abuse did not specifically report neuroimaging results from those shaken [50]. The results of the present study do not support the notion that certain medical findings are highly suggestive of shaking to the exclusion of other possible causes. The results are in agreement with those obtained by the SBU-report concluding “There is limited scientific evidence that the triad1 and therefore its components can be associated with traumatic shaking (low quality evidence)” [17].

Findings highly suggestive of blunt force head trauma, such as unexplained skull fractures, lacerations, or epidural haematoma, were found in a single case of admitted AHT by shaking. Non-specific intracranial pathology with or without RH with AHT as a possible explanation were rare. Therefore, with suspicion of child abuse, the absence or presence of findings such as SDH or RH must be interpreted with caution, and the conclusions of the social service investigation, whether initiated, should be unbiased by such findings.

Based on our results, a clinical recommendation for paediatricians and family doctors should be that a clinical suspicion of child abuse or neglect is overarching, irrespective of an abuse protocol screening findings from a CT or fundoscopy. On the other hand, screening findings of SDH or RH without any blunt force head trauma, other findings of external injury, fractures, neglect, adverse history of parenthood, psychiatric illness, substance abuse or intimate partner violence should not per se be considered as indicative for child abuse. Further, this study has clinical relevance for those clinicians who encounter children with abusive shaking witnessed or admitted at entrance. When there are no indication of a clinically negative effect and no outer signs of violence, the best option might be to give ample time for evaluating the parental competence and other safety factors.

## Conclusion

No infant subjected to shaking with or without blunt force trauma and without any possibly predisposing factors had any of the findings regarded as highly specific for AHT. Our findings imply that SDH or RH have low sensitivity for AHT, entailing a risk for false negatives if these features are believed to have a high negative predictive value. The results also indicate that isolated shaking may cause intracranial haemorrhage with or without RH in vulnerable infants.

## Supporting information

S1 Table Supplementary case description of the cases (n = 36). (DOCX)
