# Expression of Concern: Medical findings and symptoms in infants exposed to witnessed or admitted abusive shaking: A nationwide registry study

**DOI:** 10.1371/journal.pone.0306433

**Published:** 2024-06-27

**Authors:** The PLOS ONE Editors

After this article [1] was published, concerns were raised about the suitability of the study design to support the conclusions, the contextualization of the study, and the stated clinical implications. Specifically:

- The use of a retrospective study design cannot test causality, specifically the hypothesis that shaking causes subdural hemorrhage (SDH), retinal hemorrhage (RH), encephalopathy, rib fractures or classical metaphyseal lesions (CMLs). The study also does not include statistical tests for association between shaking and these injuries. Therefore, the study cannot draw any conclusions about the likelihood of abusive head trauma (AHT) in the presence of SDH or RH, as discussed in paragraphs four, seven and eight of the Discussion and in the Clinical implications section.
- The Introduction focuses on controversies surrounding the validity of a positive diagnosis of AHT based on the Duhaime algorithm [2], and does not discuss the contribution of this study [1] which may not be able to inform this debate.
- The limitations do not sufficiently indicate the assumptions made by the authors.

These concerns were investigated in consultation with members of the *PLOS ONE* Editorial Board who considered that some of the conclusions as they are stated in [1] are not supported. One member of the Editorial Board further stated that the discussions presented in [1] could imply that infant shaking/AHT does not cause injuries such as SDH and RH. They stated that [1] is not a cause-and-effect study and does not include statistical analyses. Additionally they advised that [1] should be considered a descriptive study, but it does provide evidence that shaking that is perceived as violent may not always result in demonstrable medical findings.

In response to the above concerns, the corresponding author indicated that conclusory statements in the article were not intended to indicate that shaking cannot give rise to intracranial and retinal hemorrhages, injuries to the brain tissue, fractures, and skin abrasions or bruising. The corresponding author also stated that the study conveys that the results indicate that shaking that is perceived as violent does not always result in any demonstrable medical findings. Given this, they state that the absence of findings should not be interpreted as the absence of an abuse incident, which can be important, as a supposition that shaking always leads to demonstrable injuries can lead to the risk of an investigation into abuse being stopped prematurely.

In addition, the first author provided the following corrections:

- The Conclusions section of the Abstract is corrected to: “The present findings indicate isolated shaking of a healthy infant can occur without leading to acute SDH or RH.”
- The first sentence of the sixth paragraph of the Introduction is corrected to: “In this study, we investigated the occurrence of SDH, RH, encephalopathy, rib fractures, or CMLs after shaking using a definition of physical abuse by shaking that is independent of medical findings or symptoms, i.e., not compromised by circularity.”
- The third sentence of the Circumstances defining AHT section of the Results is corrected to: “One case was filmed by the father with the mother as the perpetrator. Five cases were witnessed by strangers or neighbours.”
- The sixth sentence of the first paragraph of the Case 9 section of the Results is moved after the sixth sentence of the first paragraph of the Case 8 section of the Results.
- The first and second sentence of the Principal findings section of the Discussion are corrected to: “In earlier studies, shaking or combined shaking/blunt trauma was wholly or partly inferred from SDH, RH, seizures, apnoea, and long bone fractures. In our study, none of the infants with reported isolated shaking had any of these findings.”
- The second sentence of the sixth paragraph of the Principal findings section of the Discussion is corrected to: “Thus, our results do not provide evidence to support the appearance of rib fractures because of thorax compression or CMLs from acceleration and deceleration forces during shaking, as Kleinman et al. had proposed [4].” [3]
- The following is added to the third sentence of the first paragraph of the Strengths and limitations section of the Discussion: “and it is not possible to confirm the accuracy or severity of reported shaking incidents, especially in the absence of video footage”.
- The following is added between the second and third paragraphs of the Strengths and limitations section of the Discussion: “Analyses are only informed by data available from medical records. A medical workup—including laboratory tests—and a multi-disciplinary discussion would not be initiated in the absence of medical findings; any follow-up in cases with no medical findings would be conducted by the social authority, but such follow-up cannot be studied on the basis of medical records.”
- The first sentence of the first paragraph of the Clinical implications section of the Discussion is corrected to: “To our knowledge, this is the first study evaluating the occurrence of SDH and RH from prior witnessed or admitted physical abuse by shaking.”
- The third sentence of the first paragraph, and the second sentence in the third paragraph in the Clinical implications section of the Discussion are removed.
- The second sentence of the Conclusion is corrected to “Our findings imply that SDH or RH may have low sensitivity for isolated shaking, entailing a risk for false negatives if these features are believed to have a high negative predictive value.”
- The third sentence of the Conclusion is corrected to “The results also indicate that intracranial haemorrhage with or without RH may occur in vulnerable infants with a history of shaking.”

The *PLOS ONE* Editors issue this Expression of Concern to notify readers of the above concerns about the study design and interpretation, and that sections of the Discussion and Clinical implications should be interpreted with caution.

## References

[1] Thiblin I, Andersson J, Wester K, Wikström J, Högberg G, Högberg U (2020) Medical findings and symptoms in infants exposed to witnessed or admitted abusive shaking: A nationwide registry study. PLoS ONE 15(10): e0240182. doi: 10.1371/journal.pone.0240182 33048994 PMC7553301

[2] Duhaime AC, Alario AJ, Lewander WJ, Schut L, Sutton LN, Seidl TS, et al. Head injury in very young children: mechanisms, injury types, and ophthalmologic findings in 100 hospitalized patients younger than 2 years of age. Pediatrics. 1992;90(2 Pt 1):179–85. . 1641278

[3] Kleinman PK, Marks SC, Adams VI, Blackbourne BD. Factors affecting visualization of posterior rib fractures in abused infants. AJR Am J Roentgenol. 1988;150(3):635–8. doi: 10.2214/ajr.150.3.635 pmid:. 3257621

