# Supplementary material for: Medical findings and symptoms in infants exposed to witnessed or admitted abusive shaking: A nationwide registry study
Source: PLoS One. 2020 Oct 13;15(10):e0240182. doi: 10.1371/journal.pone.0240182 (PMC7553301; doi:10.1371/journal.pone.0240182)
Supplement: S1 Table — (DOCX) [file pone.0240182.s001.docx]

| **Case** | **Sex** | **Age** | **Term** | **Delivery** | **Neonatal conditions** | **Reported physical abuse** | **Symptoms and physical findings of external examination** | **Findings of brain imaging, whole body x-ray and fundoscopy** |
| --- | --- | --- | --- | --- | --- | --- | --- | --- |
| **Physical abuse corroborated by independent observation** | | | | | | | | |
| 1 | Male | 10 | Term | Normal vaginal | None | Filmed forceful shaking, 25 cycles | None | None |
| 2 | Male | 33 | Term | Normal vaginal | Laryngospasm and apnoea at 7th day of life | Neighbour witnessed shaking going on for an “eternity” through window | None | None |
| 3 | Female | 13 | Preterm (w 36) | Normal vaginal | None | Neighbour witnessed forceful shaking not further described | None | None |
| 4 | Male | 29 | Term | Normal vaginal | None | Neighbours witnessed forceful shaking not further described | None | None |
| 5 | Male | 8 | Term | Normal vaginal | None | Unrelated person witnessed caregiver lifting up and shaking the infant for a short moment outdoors | None | None |
| 6 | Female | 7 | Term | Normal vaginal | None | Unrelated persons witnessed caregiver lifting up and shaking the infant forcefully outdoors | None | None |
| **Admitted physical abuse** | | | | | | | | |
| 7 | Female | 10 | Term | Acute caesarean | None | Admitted and witnessed shaking at two occasions; 4 weeks old and 2 months; also put hand over mouth on at least two other occasions | Bruises on face | None |
| 8 | Male | 11 | Preterm (w 33) | Normal vaginal | Neonatal icterus. Episodes of prolonged apnoea during first week of life | Admitted forceful shaking at several occasions, followed by throwing the baby into a stroller on the last occasion in connection with seeking care | Drowsy, not unconscious;bruises on both shoulders | Acute subarachnoid haemorrhage; Acute and chronic subdural haemorrhage; Suspected cortical vein thrombosis; Non-specific white matter changes; Extensive bilateral retinal haemorrhages |
| 9 | Male | 17 | Term | Inducedvaginal | None | Admitted forceful shaking by babysitter followed by rocking when holding thorax | Drowsy, not unconsciousSeizuresVomiting | Thin bilateral acute subarachnoid haemorrhage; Bilateral chronic subdural haemorrhage; Left parietal and temporal bone fractures of unclear age |
| **Physical abuse by caregiver witnessed by the other caregiver** | | | | | | | | |
| 10 | Male | 15 | Term | Normal vaginal | None | Partner witnessed shaking, 4-5 cycles | None | None |
| 11 | Male | 29 | Term | Normal vaginal | None | Partner witnessed forceful shaking on approximately 10 occasions between ages 4 and 7 months | None | None |
| 12 | Male | 3 | Term | Acute caesarean | Small for gestational age. Signs of damage on fascial nerve | Partner witnessed short episodes of shaking when holding the thorax | None | None |
| 13 | Female | 15 | Term | Normal vaginal | None | Partner witnessed shaking when holding the thorax, yelling intent to kill the baby and the partner | None | None |
| 14 | Female | 20 | Term | Normal vaginal | Cephalohaematoma | Partner witnessed shaking not further specified at several occasions; also pressure at abdomen and hitting buttock | None | None |
| 15 | Female | 48 | Term | Normal vaginal | None | Partner witnessed repeated physical abuse by pulling limbs, applying pressure on abdomen and shaking not further specified at two occasions | None | None |
| 16 | Male | 15 | Term | Normal vaginal | None | Partner witnessed shaking not further specified on 4-5 occasions | Three episodes of vomiting | None |
| 17 | Male | 9 | Term | Normal vaginal | None | Partner witnessed forceful shaking with head bumping back and forth on approximately 8 occasions since age 2 weeks | None | None |
| 18 | Male | 7 | Term | Normal vaginal | None | Partner witnessed shaking, not further specified | None | None |
| 19 | Female | 27 | Term | Normal vaginal | None | Partner witnessed kicking, putting under water, and shaking upside down while holding in feet | None | None |
| 20 | Male | 20 | Term | Normal vaginal | None | Partner witnessed forceful shaking on several occasions | Vomiting in connection with shaking | Healing fracture left collarbone |
| 21 | Female | 15 | Term | Acute caesarean | Small for gestational age | Partner witnessed shaking, not further specified | None | None |
| 22 | Female | 27 | Term | Normal vaginal | None | Partner witnessed forceful shaking when holding thorax, followed by shaking baby stroller making the baby fall out of it | None | None |
| 23 | Female | 11 | Term | Normal vaginal | None | Partner witnessed repeated shaking and hitting on back since age 3 weeks | None | None |
| 24 | Female | 23 | Term | Normal vaginal | None | Partner witnessed forceful shaking 2 weeks before seeking care | None | None |
| 25 | Female | 11 | Term | Normal vaginal | None | Partner witnessed several episodes of “hysterical shaking”, making the head going back and forth, last time one week before seeking care | None | None |
| 26 | Male | 36 | Term | Normal vaginal | None | Partner witnessed shaking when holding onto shoulders, making the head go back and forth, followed by deliberately dropping the infant on the floor from approximately 30 cm height with back head impact | None | None |
| 27 | Female | 16 | Term | Normal vaginal | None | Partner witnessed forceful shaking when holding thorax, no further details | None | None |
| 28 | Female | 36 | Term | Normal vaginal | None | Partner witnessed forceful shaking followed by throwing/slamming the baby into a bed | Soft tissue swelling over left parietal bone | Sclerotic left parietal bone, possibly old fracture |
| 29 | Female | 35 | Term | Vacuum extraction | None | Partner witnessed shaking, no further details | None | None |
| 30 | Female | 6 | Term | Vacuum extraction | Small for gestational age | Partner witnessed forceful shaking followed by throwing the baby face down into a bed | None | None |
| 31 | Female | 10 | Term | Normal vaginal | None | Partner witnessed forceful pulling out of baby seat, followed by shaking and slapping (not further specified) | None | None |
| 32 | Female | 46 | Term | Normal vaginal | None | Partner witnessed forceful shaking at several occasions; also kicking at the baby when in walker, making the infant hit a wall;unclear when last shaking occurred | None | None |
| 33 | Male | 27 | Term | Normal vaginal | None | Partner witnessed forceful shaking at one occasion | None | None |
| **Other circumstances of corroboration of physical abuse** | | | | | | | | |
| 34 | Male | 12 | Term | Normal vaginal | None | Mother witnessed shaking by acquaintance, no further details | None | None |
| 35 | Female | 14 | Term | Normal vaginal | None | Shaking according to anonymous witness, no further details | None | None |
| 36 | Female | 3 | Term | Normal vaginal | None | Admitted shaking according to medical records, no further information | None | Two retinal haemorrhages in one eye |
